# Analysis of Protein Interactions at Native Chloroplast Membranes by Ellipsometry

**DOI:** 10.1371/journal.pone.0034455

**Published:** 2012-03-29

**Authors:** Verena Kriechbaumer, Alexei Nabok, Mohd K. Mustafa, Rukaiah Al-Ammar, Anna Tsargorodskaya, David P. Smith, Ben M. Abell

**Affiliations:** 1 Biomedical Research Centre, Sheffield Hallam University, Sheffield, United Kingdom; 2 Materials and Engineering Research Institute, Sheffield Hallam University, Sheffield, United Kingdom; University of Connecticut, United States of America

## Abstract

Membrane bound receptors play vital roles in cell signaling, and are the target for many drugs, yet their interactions with ligands are difficult to study by conventional techniques due to the technical difficulty of monitoring these interactions in lipid environments. In particular, the ability to analyse the behaviour of membrane proteins in their native membrane environment is limited. Here, we have developed a quantitative approach to detect specific interactions between low-abundance chaperone receptors within native chloroplast membranes and their soluble chaperone partners. Langmuir-Schaefer film deposition was used to deposit native chloroplasts onto gold-coated glass slides, and interactions between the molecular chaperones Hsp70 and Hsp90 and their receptors in the chloroplast membranes were detected and quantified by total internal reflection ellipsometry (TIRE). We show that native chloroplast membranes deposited on gold-coated glass slides using Langmuir-Schaefer films retain functional receptors capable of binding chaperones with high specificity and affinity. Taking into account the low chaperone receptor abundance in native membranes, these binding properties are consistent with data generated using soluble forms of the chloroplast chaperone receptors, OEP61 and Toc64. Therefore, we conclude that chloroplasts have the capacity to selectively bind chaperones, consistent with the notion that chaperones play an important role in protein targeting to chloroplasts. Importantly, this method of monitoring by TIRE does not require any protein labelling. This novel combination of techniques should be applicable to a wide variety of membranes and membrane protein receptors, thus presenting the opportunity to quantify protein interactions involved in fundamental cellular processes, and to screen for drugs that target membrane proteins.

## Introduction

The importance of studying membrane proteins is highlighted by the fact that almost half of the top-selling drugs target membrane proteins [1]. Although membrane-associated events are vital for many cellular processes, their study is complicated by the technical difficulty of monitoring these interactions in lipid environments. Current biochemical and biophysical methodology to study membrane-protein interactions requires isolation of the protein followed by reconstitution into a lipid environment, and is hindered by issues of purification and correct protein folding. As such, few techniques exist to study specific membrane-protein interactions *in situ*. An important system for quantifying protein interactions is surface plasmon resonance (SPR), and this technology has been adapted for membrane interactions by the use of supported bilayers [2]. However, the deposition of membranes is technically challenging, and the sensitivity of SPR becomes limiting for native membranes due to the lower density of target receptors. This becomes a greater problem if additional cushions are added to lift the bilayer away from the surface, which is required to maintain the fluidity of proteins within the bilayer. The development of techniques to investigate how proteins and protein complexes interact with native membranes is therefore critical, and will have direct impact on our understanding of diseases and the development of new therapeutics.

The optical method of spectroscopic ellipsometry in its total internal reflection mode (TIRE) combines spectroscopic ellipsometry and SPR and provides high sensitivity for the bio-detection of molecular interactions at a surface [3], [4]. The basis of TIRE is the detection of a change in the polarization of reflected light; in contrast to SPR which is dealing with the intensity of reflected light TIRE provides measurements of two parameters Ψ and Δ related to the amplitude and the phase of polarized light, respectively. TIRE has been successfully applied to analyze interactions between antibodies and various ligands [3], including the analysis of low molecular weight analytes such as pesticides [5] and mycotoxins with detection levels as low as 0.1 ng/ml [6]. TIRE has also been used to determine the binding affinity of the molecular chaperone Hsp70 for a soluble form of its receptor OEP61 bound to a gold surface, and was sensitive enough to discriminate between the binding affinities of closely related Hsp70 isoforms [7], [8]. This well characterized interaction [7] between Hsp70 and OEP61 is exploited here to measure specific protein-protein interactions at a native membrane.

The chaperones Hsp70 and Hsp90 are important for protein structures, in high temperature conditions and other cellular stresses, and degradation of misfolded proteins. Furthermore, cytosolic chaperones such as Hsp70 or Hsp90 are involved in protein targeting and interact with freshly translated proteins [9] preventing precursor aggregation. The recent finding of chaperone receptors in plants, such as Toc64 and OEP61 at the chloroplast outer envelope [11], [8], and mtOM64 [10] at the mitochondrial membrane, points towards a role for chaperones in organellar protein targeting. Hsp70 has been shown to be important for protein targeting to the ER [11] and mitochondria [12], and chloroplast membrane bound proteins are transported to Toc64 by Hsp90 [13]. The chloroplast chaperone receptors OEP61 and Toc64 are considered a model system here as they display typical receptor characteristics, including ligand specificity and membrane anchoring. Their low abundance provides a realistic test of the potential for the TIRE system to detect interactions of a wide range of membrane receptors.

**Figure 1:**
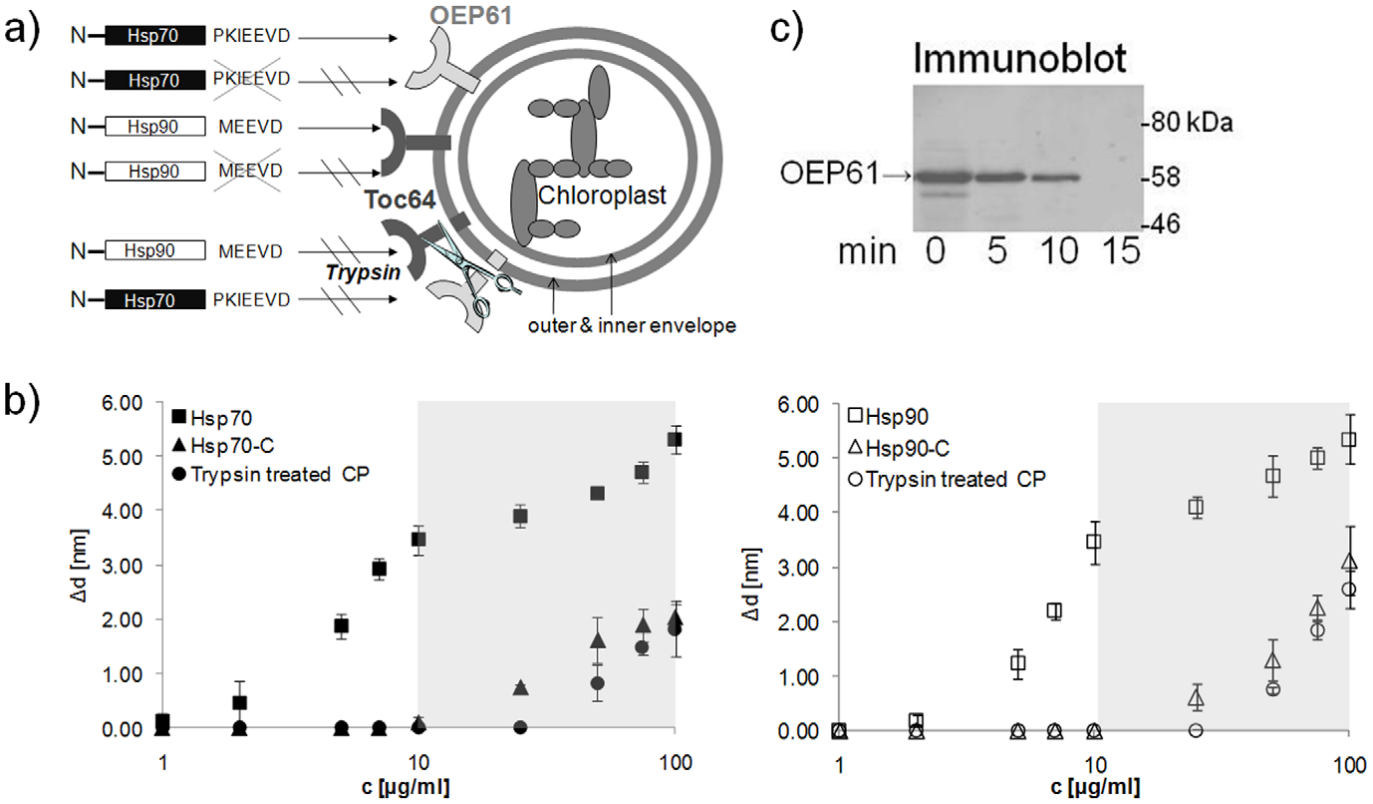
Chaperone binding to receptors at the chloroplast membrane. A) Scheme illustrating chaperone-receptor interactions. Binding of full length Hsp70 and Hsp90 chaperones to chaperone receptors at the chloroplast outer envelope is shown. Truncated chaperone sequences and trypsin digestion of membrane proteins used as negative controls are indicated. B) Specific binding of chaperone proteins to native chloroplast membranes. Calibration curves (layer thickness increment Δd vs. increasing chaperone concentrations) for Hsp70 (left, ▪) or Hsp90 (right, □) binding to native chloroplast membranes deposited on a gold surface. Truncated chaperone proteins (Hsp70-C and Hsp90-C; ▴ and ▵) and chloroplasts treated with the protease trypsin (• and ○) were used to distinguish between specific and nonspecific binding. Grey background indicates the area of nonspecific binding. C) Trypsin digestion of chloroplast membranes is controlled by testing for the presence of the plastidial receptor OEP61. Following tryptic digests of chloroplast membranes for 0, 5, 10, and 15 min, immunoprecipitation of OEP61 and immunoblotting with anti-OEP61-IgG were performed. The OEP61 band and protein sizes (kDa) are indicated.

OEP61 resides in plastids throughout the plant, and selectively binds Hsp70, but not Hsp90 chaperones, via its tetratricopeptide repeat (TPR) domain [7], [8]. Since this TPR domain is displayed outside of the membrane and chaperone-receptor interaction takes place outside of the membrane such interactions are not expected to be influenced by membrane fluidity or tension. Preferential binding of precursors destined for chloroplasts indicates that OEP61 plays a role in protein targeting, and may work in concert with other plastidial chaperone receptors such as Toc64, which binds Hsp90 via its TPR domain [14]. Both OEP61 and Toc64 are anchored to the chloroplast outer envelope by transmembrane domains (Fig. 1A), and are predicted to interact with other membrane components to facilitate protein targeting. Indeed, Toc64 was found to be a transient component of the translocon complex facilitating protein import of chaperone bound precursor proteins into chloroplasts [13], [15]. Thus, TIRE analysis at native membranes potentially supports such membrane-dependent interactions.

We show here that TIRE can be used to obtain binding affinities of specific chaperone-receptor interactions on native chloroplast membranes without the need to purify the target receptor.

## Materials and Methods

### Chloroplast membrane preparation

Chloroplasts from pea were purified as previously described [8]. This method was shown to result in intact, import-competent chloroplasts with receptors present on the outer envelope [8]. 3 g pea leaves were cut into small pieces (∼2 mm×2 mm), transferred into 50 ml Falcon tubes with 30 ml ice-cold grinding buffer (2 mM EDTA (pH 8.0), 1 mM MgCl_2_, 1 mM MnCl_2_, 50 mM HEPES (pH 7.5), 0.33 M sorbitol, 0.5 g/l sodium ascorbate, 1.25 g/l BSA) and homogenized for 1 min with a Polytron homogenizer (IKA T 18 basic ULTRA-TURRAX, intensity level 2). The homogenate was filtered through 4 layers of cheesecloth and centrifuged for 2 min at 3000 g and 4°C. The resulting pellet was resuspended in 0.5 ml grinding buffer using a paintbrush. After layering the pellet onto Percoll step gradient (1.5 ml 80% Percoll overlaid with 5 ml 40% Percoll), and centrifugation for 8 min at 9000 g and 4°C, the intact chloroplasts in the lower band between Percoll layers were transferred to a fresh 15 ml tube, avoiding transfer of 80% Percoll. The volume was adjusted to 5 ml with grinding buffer, centrifuged for 5 min at 2000 g and 4°C, and the pellet redissolved in 0.5 ml 1× HSM (50 mM HEPES (pH 7.5), 0.33 M sorbitol, 8.4 mM methionine). The purified chloroplasts were frozen in liquid nitrogen and stored at −80°C.

### Langmuir-Schaefer deposition

Standard microscopic glass slides (1″×1″) were coated with Cr (3 nm thick) and Au (25 nm thick) using a thermal evaporation unit (Edwards A360); these metal films were deposited without breaking the vacuum of 10^−6^ Tor. Cr improves the adhesion of Au to glass. Then the slides were incubated overnight in a 100 mM solution of cysteamine-HCl to provide a positive surface charge. A mini-Langmuir trough (KSV NIMA, Espoo, Finland) was used for the deposition of chloroplasts on gold coated glass slides. Membrane layers were created on the surface of de-ionized water (ELGA, Marlow, UK) with a resistivity ≥15 MΩ.cm. 10 µl of the chloroplast solution was dotted onto the water surface using a Hamilton syringe. For this little droplets of approximately 1 µl were formed at the tip of the Hamilton syringe and these droplets were brought into contact with the water surface of the Langmuir trough. Droplets were positioned evenly distributed over the trough surface area. A typical isotherm of chloroplast layers on the water surface is shown in Fig. S1A. A linear rise of the surface pressure (Π) upon compression demonstrates the formation of a two-dimensional solid phase of chloroplasts on the water surface. A pressure of 20 mN/m was chosen for deposition.

The Langmuir-Schaefer method of horizontal lifting [16] was used to transfer the membranes onto the slides. The slides were fixed almost horizontally (a tilt angle of few degrees improves the deposition) on the sample holder and lowered down at a low speed of 10 mm/min until touching the water surface covered with chloroplast membranes, and then the slide was lifted up again. As a result, a negatively charged layer of chloroplast membranes was attached to the positively charged surface of gold. The deposition process was monitored by recording the surface pressure (Π) during deposition (Fig. S1B). Analysis of a large number of depositions (up to 40) showed that during each deposition step a chloroplast film with an average surface area of 6.39±0.88 (SE) cm^2^ was transferred. This corresponds remarkably well to the slide area of 6.45 cm^2^ and proved that the transfer ratio was close to unity; in other words, chloroplast membranes were transferred onto solid substrates without distortion.

For proteolytic digestion chloroplast membranes were incubated with 0.2 U/µl trypsin (Sigma, Dorset, UK) for 15 min at 20°C. Before addition of chaperones trypsin was inactivated by applying soybean trypsin inhibitor (Sigma, Dorset, UK) at 1 mg/ml for 15 min.

### Immunoblotting

Prior to immunoblotting, OEP61 was immunoprecipitated from 20 µl trypsin-digested chloroplast membranes. Samples were precleared by adding four volumes of TXIP buffer (10 mM Tris-HCl (pH 7.5), 140 mM NaCl, 1 mM EDTA, 1% Triton X-100 and 2 mM PMSF) and 0.1 volume pansorbin (Calbiochem, Darmstadt, Germany) and incubated at 4°C for 30 min. The mixture was centrifuged at 14300 g for 10 min at 4°C to pellet the pansorbin, and the supernatant was incubated with anti-OEP61 IgG (custom-made by Eurogentec, Brussels, Belgium) at a 1∶1000 dilution, followed by overnight incubation at 4°C. Protein A-Sepharose beads (1∶100 dilution; Sigma, Dorset, UK) were added and incubated for 1.5 h at 4°C. The beads were washed four times with 1 ml of TXIP buffer and 40 µl denaturing loading buffer was added. 20 µl of the eluted proteins were separated by SDS-PAGE (12% separation gel) and transferred to PVDF membranes (Millipore, Billerica, MA) according to the manufacturers' instructions. Primary antibodies were used at 1∶1000 for the anti-OEP61-IgG serum. Secondary goat anti-rabbit IgG labeled with red-fluorescent IRDye 680 (LI-COR Biosciences, Lincoln, NE) were used at 1∶3000, and fluorescence was detected using the ODYSSEY Infrared imaging system (LI-COR Biosciences, Lincoln, NE).

### Heterologous protein expression and purification

Clones for Hsp70-1 (At5g02500) and Hsp90-2 (At5g56030) were obtained as cDNAs in plasmids from The Arabidopsis Information Resource (TAIR, Stanford, CA). The coding sequences as well as C-terminally truncated forms were amplified using the following primer combinations introducing *Nde*I and *BamH*I restriction sites for Hsp70 and only B*amH*I for Hsp90, respectively:

Hsp70for ctttggcagatctacccatatgtcgggtaaaggag, Hsp70rev ttaaggatccttagtcgacctcctc,

Hsp70rev-C ttaaggatccttatccagcaccgcc, Hsp90for: ctttggcagatctacccatatggcggacgctgaaac,

Hsp90rev: taaacatatgttagtcgacttcctc, Hsp90rev-C taaacatatgttacatcttgctaccttcgg.

The obtained PCR products were cloned into pET16b or pET28b expression vectors containing 6-His-tags (Novagen, Madison, WI) and proteins were heterologously expressed in T7 Express Iq *E.coli* cells (New England Biolabs, Ipswich, MA).

Bacterial cells were harvested by centrifugation at 8000 g for 8 min, resuspended in lysis buffer (50 mM NaH_2_PO_4_, 300 mM NaCl, 10 mM imidazol, pH 8.0), and treated with lysozyme (1 mg/ml) for 30 min on ice. The lysate was sonicated three times at 200 W for 10 s and was centrifuged at 10000 g for 30 min at 4°C. His-tagged proteins were purified under native conditions using Ni-NTA agarose according to the manufacturer's manual (Qiagen, Crawley, UK), dialyzed against 50 mM Tris-HCl, pH 8.0, and their His-tag cleaved using AcTEV protease (Invitrogen, Paisley, UK) according to the manufacturers' instructions.

### TIRE measurements and data fitting

TIRE measurements were performed using the J.A. Woollam spectroscopic ellipsometer M2000 operating in the spectral range of 370–1000 nm, and exploiting the rotating compensator principle. The additional element in TIRE is a 68° trapezoidal glass prism through which the light is coupled into thin metal (Au) film deposited on a glass slide. Index matching fluid is used for optical contact between the prism and glass slide. The reaction cell with the volume of 0.2 ml is positioned underneath the gold layer. Inlet and outlet tubes allow the injection of required solutions into the cell to perform adsorption of different molecules on the surface.

Cr/Au slides with deposited chloroplast membranes were washed with at least 20 cell volumes of 100 mM Tris-HCl, pH 8.0, before flushing the cell with chaperones in 100 mM Tris-HCl, pH 8.0 in increasing concentrations (from 1 ng/ml to 100 µg/ml) with 15 min incubation time. The cell was rinsed after every deposition step by purging 20 times the cell volume with 100 mM Tris-HCl, pH 8.0.

Spectroscopic ellipsometry provides the spectra of two ellipsometric parameters Ψ and Δ, which are related to the ratio of the amplitudes and the phase shift of p- and s- components of polarized light, respectively. The ability to record spectra of the parameter Δ distinguishes TIRE from conventional SPR. Δ is 10 times more sensitive than Ψ to small changes in the optical density of adsorbed molecular layers, which constitutes the main advantage of using TIRE compared to SPR [3], [4].

Two types of ellipsometric measurements were performed: (i) TIRE single spectra scans were recorded after completion of every adsorption step in a standard buffer solution (100 mM Tris-HCl, pH 8.0); (ii) dynamic TIRE spectral measurements, in which a number of TIRE spectra were recorded during every adsorption step. Since the refractive index of injected solutions may vary, the evaluation of the thickness and optical constants is not strictly correct for dynamic scans. Dynamic TIRE measurements can be used, however, to analyze the kinetics of adsorption or binding reactions. Single spectra scans performed in the same buffer solution in steady-state conditions after completion of adsorption were thus suitable for TIRE data fitting.

Software provided by J.A. Woollam Ltd [17] allowed the modeling of the reflection system and subsequent evaluation of the thickness and refractive index of adsorbed molecular layers by comparing the experimental and theoretical values of Ψ and Δ and minimizing the error function. A four-layer model, typically used in our TIRE measurements, is shown in Table 1. The thickness (*d*) and complex refractive index (*n-ik*) dispersion for a gold layer was evaluated first by fitting TIRE spectra recorded on the Cr/Au surface using Fresnel equations (Table 1); then the obtained parameters were kept fixed during further fittings on the same sample. A principle limitation of ellipsometry and SPR is that the simultaneous evaluation of *d* and *n* of thin (less then 10 nm) dielectric films is impossible and either *d* or *n* must be fixed during the fitting. Here the refractive index is kept fixed and all the changes in the adsorbed layer are associated with the thickness. This is close to reality since all bio-organic substances have similar refractive indices of about 1.42 at 630 nm [18].

**Table 1:** Four-layer TIRE model.

Layer	Thickness *(d)*, refractive index *(N = n-jk)*	Comments
BK7 glass	*d*≥1 mm, *n* = 1.515 (at 633 nm), *k* = 0 dispersion function *n*(λ) for BK7 is from J.A. Woollam database [13].	Parameters are fixed.
Cr/Au	*d* is typically of 27–32 nm, Initial dispersion function for Au is from J.A. Woollam database [13].	Effective optical dispersion parameters *N*(λ) for Cr/Au layer were determined by fitting the data for bare Au surface; then d and *N*(λ) were fixed in consecutive data fitting.
Molecular layer	*d* is variable (subject of fitting); *n*(λ) is described by Cauchy model from J.A. Woollam database [13]. 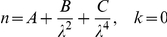	*n* is a fixed parameter (*n* = 1.42 at 633 nm) with *A* = 1.39, *B* = 0.01, *C* = 0
Water	*d*≥1 mm, *n* = 1.42 (at 633 nm), *k* = 0 dispersion function *n*(λ) for water is from J.A. Woollam database [13].	Parameters are fixed.

### Evaluation of the association constant from adsorption kinetics

**Figure 2:**
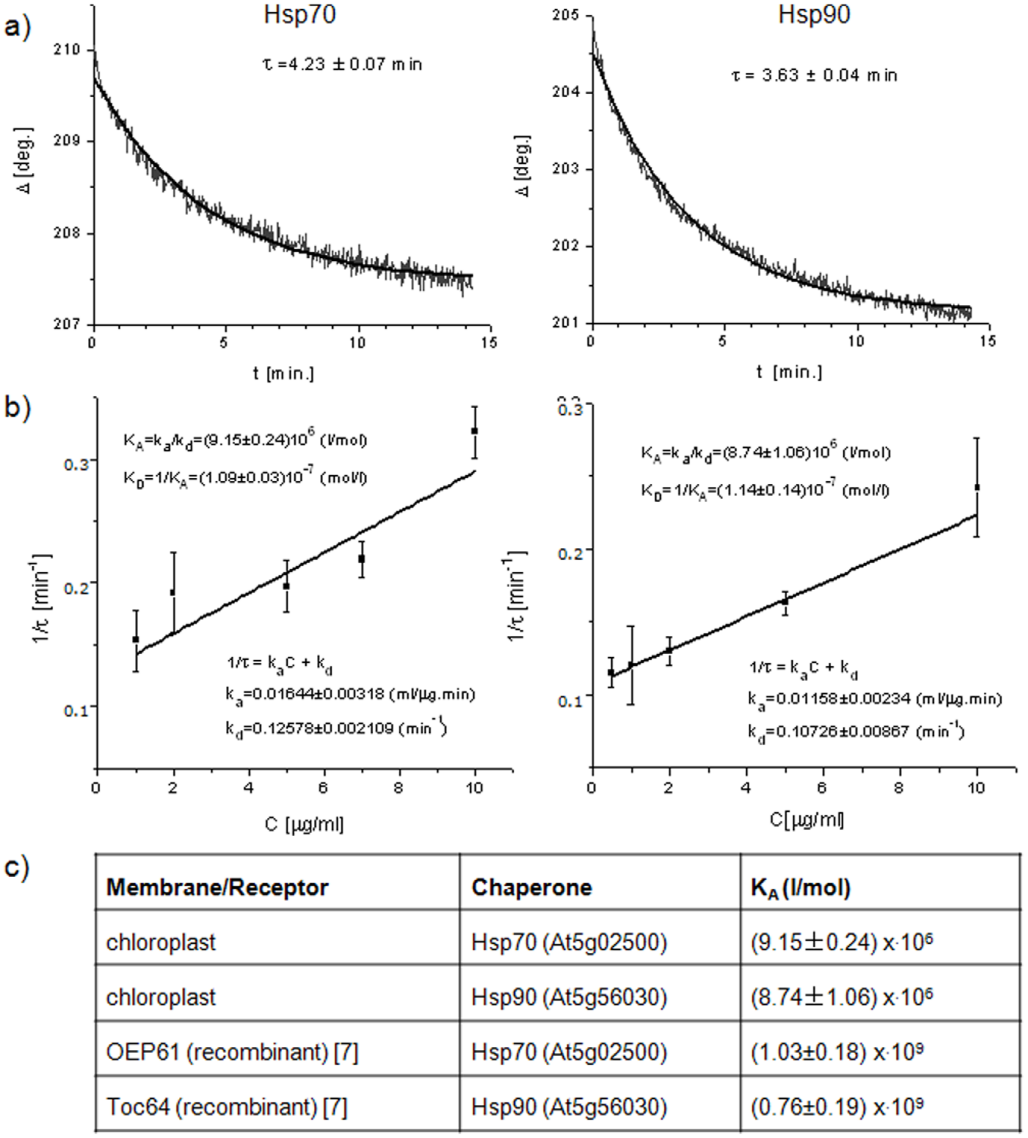
Dynamic measurements and association constants. A) Typical time dependencies of the parameter Δ during binding of Hsp70 (left) and Hsp90 (right). The time dependences Δ(τ) at 700 nm during chaperone binding to chloroplast membranes are fitted to a single exponential decay function. Values of the time constants (τ) obtained by fitting are given in the graph. B) Graphical evaluation of the association (*K_A_*) or affinity (*K_D_*) constants of Hsp70 and Hsp90 to chloroplasts. The reciprocal values of the time constant (1/τ) are plotted against the chaperone concentration (*C*), fitted to a linear function 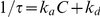, and the rates of adsorption (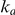) and desorption (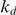), respectively, are calculated from the gradient and intercept. The association constant K_A_ is calculated as a ratio of 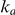 and 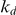 (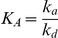) and the obtained values of *K_A_* and *K_D_* are given on the graphs. C) Calculated association constants for binding of Hsp70 or Hsp90 chaperones to native chloroplast membranes are given in comparison to association constants for chaperone interaction with the recombinantly expressed receptor proteins OEP61 and Toc64 [7].

For the evaluation of association (or affinity) constants a standard procedure of adsorption kinetics [6], [19] was used. The interaction between receptor and chaperone was monitored by observing changes in the sensor response. First, the time constants (τ) were determined from the time dependences of the ellipsometric parameter Δ obtained by dynamic TIRE measurements at different chaperone concentrations. Fig. 2A shows typical time dependences of Δ recorded during binding Hsp70 and Hsp90 chaperones and the fitting to a first order exponential decay function 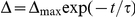. The reciprocal values of the time constants (*1/τ*) were plotted against the concentration of chaperones (*C*) and fitted to the linear function 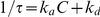 (Fig. 2B). The rates of adsorption (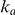) and de-sorption (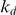), respectively, were calculated from the gradient and intercept of this line. The association and affinity constants defined as 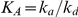 and 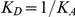, respectively, can therefore be evaluated. Data points in Fig. 2B were obtained by averaging the results of several independent (n>3) TIRE kinetic measurements. The obtained values of the gradient (*k_a_*) and intercept (*k_d_*) as well as the resulted values of *K_A_* and *K_D_* are given on the respective graphs. The standard deviations were calculated as 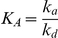.

## Results and Discussion

### Steady-state measurements

Chloroplasts were isolated by gradient centrifugation, resuspended in Tris-HCl buffer and deposited on chromium/gold coated glass slides via Langmuir-Schaefer films. Previous studies have shown that these chloroplasts are intact and import competent, with the outer envelope receptor OEP61 facing the cytosol [8]. We cannot exclude the existence of a minor population of chloroplast membranes that is fragmented and inverted, which would not present receptors to the test solution, but this is not expected to reduce total binding significantly. These slides were fitted into the ellipsometer cell and flushed with recombinantly expressed chaperone proteins. TIRE spectra were recorded in steady-state conditions after completion of adsorption of chaperones in progressively increasing concentrations (Fig. 1B). Fitting the obtained TIRE spectra allowed the evaluation of the layer thicknesses and therefore the level of binding after each adsorption step. Layer thickness resolution lies in the range of 0.01 nm which is sufficient for detection of chaperone binding responses at concentrations down to 1 ng/ml [7].

These TIRE steady-state measurements (Fig. 1B, left) show that Hsp70 binding to the deposited chloroplast membranes can be observed at concentrations of 1 µg/ml, followed by an increase in layer thickness to 10 µg/ml, which is likely to represent specific TPR-domain based binding. This is followed by further interactions at very high concentrations of Hsp70, which are likely to be non-specific and have been attributed to protein crowding effects. Hsp90 binding (Fig. 1B, right) follows similar patterns with binding starting at 2 µg/ml Hsp90 concentration followed by a steep increase in layer thickness up to 10 µg/ml Hsp90 chaperone. Hence, binding to native low-abundance receptors can be detected as Toc64 represents approximately 0.5% outer envelope proteins [20], and OEP61 represents approximately 1.5% outer envelope proteins based on semi-quantitative immunoblots [8].

Binding specificity was determined using truncated versions of chaperones (Hsp70-C, Hsp90-C) that lack a C-terminal heptapeptide (PKIEEVD for Hsp70) [21], [22] or pentapeptide (MEEVD for Hsp90) [14], [21], respectively (Fig. 1A). These amino acid sequences are known to be responsible for binding specifically to the TPR domain of the receptor [21], [22].

Binding of the truncated Hsp70-C to the membrane is first observed at the relatively high concentration of 10 µg/ml (Fig. 1B, left) followed by a steady increase in layer thickness. This increase following the same trend as Hsp70 at these higher concentrations indicates that non-specific binding occurs, and that the binding observed at concentrations above 10 µg/ml is not mediated by specific receptors. Similarly, the truncated Hsp90 (Hsp90-C) only binds membranes significantly at concentrations above 25 µg/ml (Fig. 1B, right).

To further confirm that the binding observed on the membrane is indeed specific and receptor dependent, chloroplast membranes were incubated with the protease trypsin to degrade all exposed proteins such as the TPR domains of OEP61 and Toc64 (Fig. 1A). Optimal conditions were determined using immunoblots against the receptor OEP61, and it was shown that after digestion with 0.2 U/µl trypsin for 15 min at 20°C OEP61 could not be detected anymore (Fig. 1C). For ellipsometric measurements chloroplast membranes deposited on gold slides were trypsinized using these conditions, and then the trypsin was inhibited using soybean trypsin inhibitor. Full-length Hsp70 or Hsp90 chaperones, in increasing concentrations from 1 ng/ml were applied to the receptor-depleted membranes, and their binding measured. Binding of both Hsp70 and Hsp90 was only observed above concentrations of 50 µg/ml (Fig. 1B), demonstrating that the specific binding observed with intact membranes was due to proteins exposed on the surface.

Taken together, both Hsp70 and Hsp90 chaperone proteins show very similar binding profiles with native chloroplast membranes and there is a clear discrimination between specific binding at lower chaperone concentrations between 1 and 10 µg/ml and non-specific binding at higher concentrations as occurring with the truncated chaperone versions or membranes depleted of receptor proteins. These results demonstrate that native membranes as a tissue source can be isolated and utilized such that specific protein-receptor binding events can be observed in the context of a complete membrane system and with low receptor abundance.

### Dynamic measurements and binding kinetics

To obtain binding affinities and to observe differences in the binding behavior of full-length and truncated chaperones dynamic spectral measurements were carried out during molecular adsorption. Data was recorded over 15 min following initial flushing of the membranes with chaperones, and the time dependences of the phase-depending parameter Δ at 700 nm were extracted (Fig. 2A) and used for the analysis of binding kinetics (Fig. 2B). Affinity constants K_A_ were calculated for chaperone concentrations from 1 µg/ml up to 10 µg/ml, representing the area of specific membrane binding (Fig. 2C). K_A_ values were determined as (9.15±0.24)×.10^6^ (l/mol) for Hsp70 and (8.74±1.06)×.10^6^ (l/mol) for Hsp90. For comparison Hsp70 and Hsp90 bind their co-chaperone the Hsp70-Hsp90-organising protein (Hop) with a K_A_ of 7.7×.10^5^ (l/mol) and 1.1×10^7^ (l/mol), respectively [23] providing further evidence that specific membrane interactions are being observed. The K_A_ values are two to three orders of magnitude lower than the affinity constants previously calculated for chaperones with the recombinantly expressed receptors OEP61 and Toc64 (Fig. 2C) [7], which is probably due to the change to a more native environment and unspecific binding to proteins at the native membranes resulting in a higher dissociation rate.

Although the chaperones must be binding TPR clamp receptors, and OEP61 and Toc64 are assumed to be the only known chloroplast examples, a contribution from undiscovered chaperone receptors cannot be excluded. To identify membrane-bound receptors by conventional biochemical approaches is difficult due to their low abundance and hydrophobicity. Therefore, a number of bioinformatics searches based on structural alignments [8] or TPR domain features [24] have been performed, but so far resulted in no further identification of chloroplast chaperone receptors. The TIRE system could be used to test for additional chaperone-receptor interactions by comparing chaperone affinities for wildtype *Arabidopsis* chloroplasts and chloroplasts isolated from *oep61* and/or *toc64* knockout mutants.

### Conclusions

In summary, these studies demonstrate that chloroplasts can be deposited on gold coated glass slides via Langmuir-Schaefer films, whilst retaining functionality of the resident receptors. Binding of receptors to their specific protein partner can be directly monitored using TIRE, without the need for labels. Potentially, molecular interactions at any other type of membrane, such as mitochondrial or ER membranes, could be analyzed using this combination of Langmuir-Schaefer films and spectroscopic ellipsometry and is the focus of ongoing work. As such, this technique opens many possibilities including the quantification of protein interactions involved in fundamental cellular processes, and rapid screening of drugs that target membrane proteins.

## Supporting Information

Figure S1 A) Typical Π-A diagram of chloroplast membranes on a water surface. Area compression is plotted against increase in surface pressure (Π). B) Monitoring of Π during multiple Langmuir-Schaefer depositions of chloroplast membranes.
(TIF)
