# Supplementary figures and images for: Analysis of Protein Interactions at Native Chloroplast Membranes by Ellipsometry
Source: PLoS One. 2012 Mar 29;7(3):e34455. doi: 10.1371/journal.pone.0034455 (PMC3315527; doi:10.1371/journal.pone.0034455)

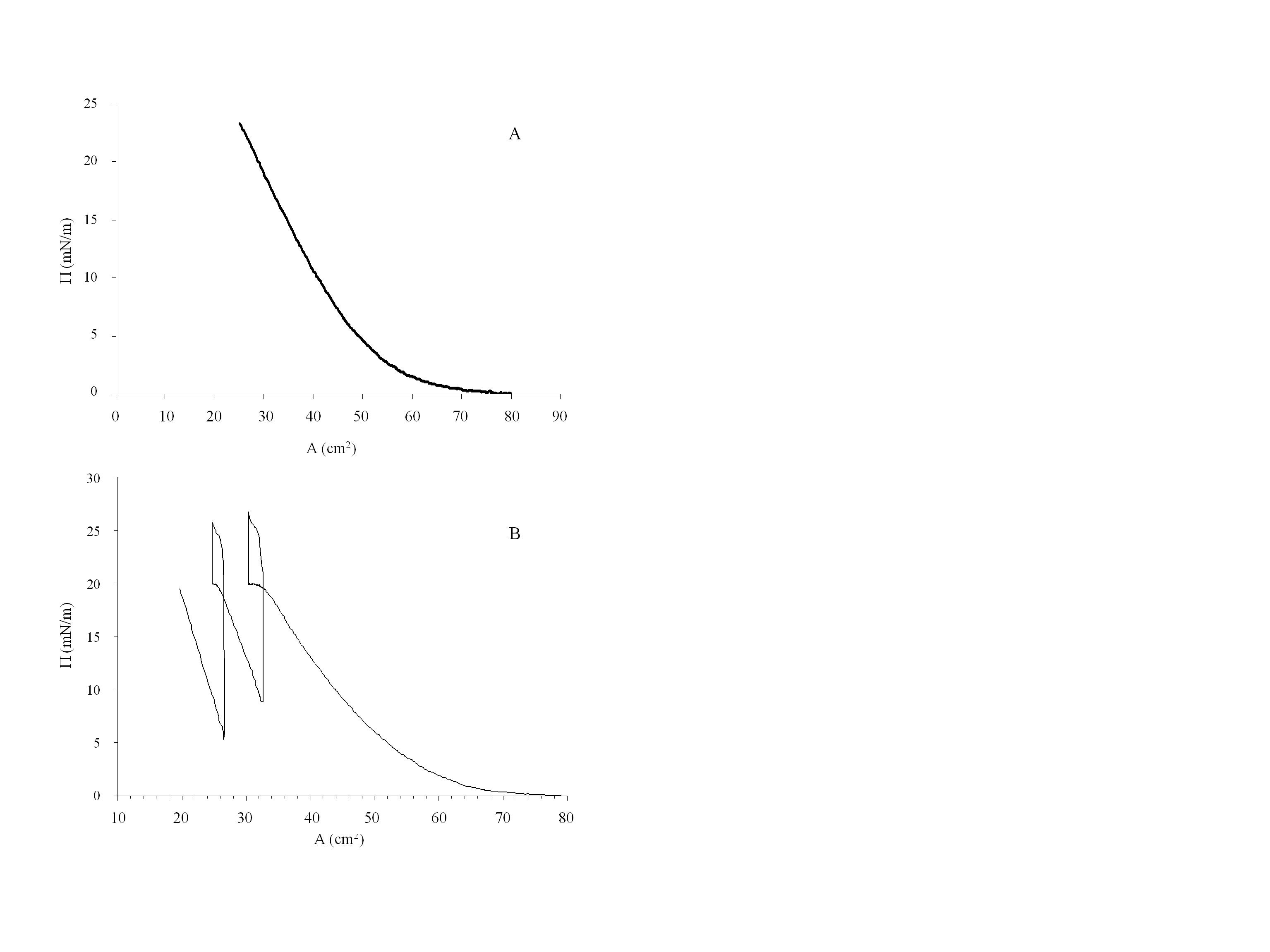

Supplement: Figure S1 — A) Typical Π-A diagram of chloroplast membranes on a water surface. Area compression is plotted against increase in surface pressure (Π). B) Monitoring of Π during multiple Langmuir-Schaefer depositions of chloroplast membranes. (TIF) [file pone.0034455.s001.tif]
